# Could maternal serum MFG-E8 level predict adverse first trimester pregnancy outcome? A preliminary study

**DOI:** 10.55730/1300-0144.5614

**Published:** 2023-01-15

**Authors:** Bircan ELÇİ, Zeynep YALÇINKAYA, Ercan TEKİN, Şükrü BAKIRCI, Cemile DAYANGAN SAYAN, Üçler KISA, Mertihan KURDOĞLU, Zehra Sema ÖZKAN, Nevin SAĞSÖZ

**Affiliations:** 1 Department of Obstetrics and Gynecology, Faculty of Medicine, Kırıkkale University, Kırıkkale, Turkey; 2 Department of Public Health, Afyonkarahisar State Hospital, Afyonkarahisar, Turkey; 3 Department of Biochemistry, Faculty of Medicine, Kırıkkale University, Kırıkkale, Turkey

**Keywords:** MFG-E8, missed abortion, pregnancy outcome, live fetus

## Abstract

**Background/aim:**

Milk fat globule-epidermal growth factor 8 **(**MFG-E8) is expressed in the endometrial epithelium and its expression increases during the implantation process. Due to this knowledge, we aimed to investigate the maternal serum MFG-E8 levels on both healthy pregnant women in the first trimester and pregnant women complicated with missed abortion and threatened abortion in the first trimester.

**Materials and methods:**

This prospective, cross-sectional study was conducted in a tertiary referral hospital, department of obstetrics between July 2020 and February 2021 after ethical committee approval. The study population was consisted of 30 healthy pregnant women (HP) in the first trimester, 30 pregnant women suffering from threatened abortion (TA) in the first trimester and 30 pregnant women suffering from missed abortion (MA) in the first trimester. Maternal serum MFG-E8 levels were analyzed with enzyme linked immunosorbent assay. Delivery and neonatal outcomes of the study population was evaluated. The continuous variables were compared among three groups with variance analysis with post hoc tests. The categorical variables were compared with chi-square and Fisher’s exact tests where applicable.

**Results:**

The mean age of the study population was 29.36 ± 5.31 years. There was no significant difference among three groups for parameters of age, body mass index, parity number, and gestational week. Despite being within normal ranges, the mean neutrophil and international normalized ratio values of the three groups showed statistically significant difference (p < 0.05). The mean maternal serum MFG-E8 levels of MA, TA, and HP groups were 270 ± 152.3, 414.7 ± 236.7, and 474 ± 222.5 ng/mL, respectively (p = 0.001). It was found that mean of MFG-E8 of the MA group was statistically significantly lower than those of the other two groups (p < 0.05).

**Conclusion:**

Although maternal serum MFG-E8 level seems to be a parameter that differ between live and nonlive pregnancies, studies with large number of cases are needed to discuss our results and to determine a cut-off value for prediction

## 1. Introduction

Spontaneous abortion is the expulsion of all or part of the embryo or fetus, along with its appendages, which has not completed the 20th gestational week or weighs less than 500 g from the uterine cavity [1]. Fifteen to twenty percent of pregnancies result in spontaneous abortion [2].

Threatened abortion, which is the most common complication of pregnancy, occurs in 15%–20% of pregnancies [3]. More than half of pregnancies in which vaginal bleeding is experienced during early pregnancy result in spontaneous abortion and pregnancy complications occur 2.2 times more than healthy pregnancies in advanced weeks of gestation [4,5].

Missed abortion accounts for approximately 15% of clinically diagnosed pregnancies [6]. Serious bleeding and coagulation disorders may occur when the products of pregnancy cannot be expelled from the uterine cavity for a long time after the intrauterine death of the embryo or fetus [7].

Milk fat globule-epidermal growth factor 8 (MFG-E8) is a glycoprotein that was first identified as a component of the milk fat globule membrane [8]. It has various functions such as clearance of apoptotic cells, cell adhesion, remodeling, angiogenesis, fatty acid absorption and immunomodulation [9–13]. MFG-E8 has also recently been associated with the implantation process [14]. It has been shown that MFG-E8 is expressed in the endometrial epithelium and its expression increases during the implantation process [15].

Especially, the endocrine and inflammatory processes occurring during implantation play an important role in the smooth continuation of pregnancy [16–19]. Problems in these processes first lead to placental invasion problems, then to first trimester bleeding with abortion risk [20]. It will make a valuable contribution to the literature to reveal whether the MFG-E8 glycoprotein can be used in the prediction of early pregnancy prognosis. Thus, in this study, we aimed to investigate the maternal serum MFG-E8 levels in healthy and complicated-first-trimester pregnant women.

## 2. Materials and methods

### 2.1. Study population

This prospective, cross-sectional study was conducted between July 2020 and February 2021 in University Hospital, Department of Obstetrics and Gynecology after the local ethical committee approval (dated 16.07.2020 and numbered 11/03). The pregnant women between the ages of 18 and 40 years old, who had a first trimester pregnancy (in the first 14 weeks of pregnancy), who agreed to participate in the study was included in the study population. The study population consisted of 30 pregnant women in the first trimester with a single fetus diagnosed with missed abortion (MA group), 30 pregnant women in the first trimester with a single fetus suffering from threatened abortion (TA group), and 30 healthy pregnant women in the first trimester with a single fetus (HP group). We had difficulty about finding patients due to COVID-19 restrictions. For the same reason, we could not evaluate plasma mfg-e8 level change according to trimesters and its placental level after delivery. The cases in which pregnant women experienced vaginal bleeding while their pregnancy was still in the first 14 weeks were considered threatened abortion. Pregnancies in which fetal cardiac activity could not be detected ultrasonographically before the 14th week of pregnancy were considered missed abortion. Healthy pregnant women were accepted as pregnant women who applied to our outpatient clinic for routine obstetric follow-up and did not have any systemic disease or obstetric problems.

Pregnant women suffering from an autoimmune disease, thyroid disease, hyperemesis gravidarum, diabetes mellitus, multiple pregnancy, coagulopathy, using anticoagulant or steroid medication, who refused to participate in the study, and who were under the age of 18 and over 40 years of age were excluded from the study. Obstetric examinations of 30 TA, 30 MA, and 30 HP women were performed. Gestational weeks were determined according to the last menstrual period of the pregnant women. Fetal biometric measurement was performed by measuring crown rump length (CRL) with abdominal ultrasound (USG) (Voluson P8, General Electrics, USA). Fetal heartbeats were observed in all of the pregnant women in the TA and HP groups. Fetal heartbeats were not observed in any of the MA groups.

BMI (body mass index) was calculated by measuring the height and weight of the pregnant women. In addition, systolic and diastolic blood pressure measurements of pregnant women at rest were made. Demographic characteristics, obstetric history, gestational week, CRL measurement, fetal heart activity, smoking and alcohol use, blood group, and systolic and diastolic blood pressure values of each pregnant woman included in the study were recorded. Also, white blood cell count (WBC), neutrophil, lymphocyte, hemoglobin, hematocrit, platelet, platelet distribution width (PDW), mean platelet volume (MPV), thyroid stimulating hormone (TSH), aspartate aminotransferase (AST), alanine aminotransferase (ALT), and international normalized ratio (INR) values during routine pregnancy follow-up and MFG-E8 values evaluated by ELISA (enzyme-linked immunosorbent assay) method in maternal serum of pregnant women were evaluated and recorded. The data of delivery week, delivery route (vaginal/abdominal), neonatal birth weight, Apgar first and fifth minute scores, and pregnancy outcomes of the TA and HP groups were collected either via hospital records or by phone call.

### 2.2. Blood sampling

Five-milliliter blood samples were taken from antecubital peripheral veins from pregnant women participating in the study with a sterile injector. Serum fractions of blood samples were separated by centrifugation, frozen at −80 °C, and stored until the assay. On the assay day, the cryo-preserved serums were removed from their environment at −80 °C and allowed to dissolve at room temperature. Serums were analyzed with the MFG-E8 ELISA kit (catalog no=201-12-4263, Sunred, Shanghai, China) according to manufacturer’s instructions. The MFG-E8 serum concentration measurement is given as ng/mL. The lowest level for measurement is 7.046 ng/mL. Intraassay for measurement CV (coefficient of variability) is <10%; interassay CV is <12% (CV%= (SD = standard deviation/mean × 100).

### 2.3. Statistical analysis

For statistical analysis, IBM SPSS Statistics 23.0 package program (IMB Corp., New York, USA) was used. There is no reference study similar to our study. Therefore, the effect size was taken as large. We calculated the sample size as 84 for 90% power, an error of 0.05 and large effect size. Due to possible drops, we designed the study as 30 pregnant women in each group. In descriptive statistical analyses; mean ± standard deviation and median (minimum-maximum) were given for continuous variables according to data distribution normality. Number and percentage (%) was given for categorical variables.

The distribution of continuous variables was evaluated with the Kolmogorov–Smirnov test and parametric or nonparametric tests were preferred according to the test result. One-way ANOVA and post hoc Tukey test was used for comparison of parametric data among groups. The Kruskal–Wallis test and the Mann–Whitney U test were used to compare nonparametric data. The categorical variables were compared with chi-square and Fisher’s exact tests where applicable. ROC analysis was used to determine whether the MFG-E8 value has a predictive potency for missed abortion prediction. Spearman correlation analysis was performed to investigate the possible relation between MFG-E8 levels and neonatal birth weight, delivery week, Apgar 1 and 5 scores. Statistical significance level was accepted as p < 0.05.

## 3. Results

The demographic and clinical characteristics of the study population are presented in Table 1. The BMI of the pregnant women participating in the study was 25.79 ± 4.54 kg/m^2^. The mean ages of all pregnant women who participated in the study, as well as the 1st trimester missed abortion, abortus imminens, and healthy pregnancy groups were 29.36 ± 5.31, 29.83 ± 6.21, 29.00 ± 4.48, and 29.23 ± 5.24, respectively. The median week of gestation was 9.4 (6.1–13.7), 9.4 (6.7–13.5), 7.8 (6.1–13.7), and 10.2 (6.1–13.5) weeks in all pregnant women, missed abortion, abortus imminens, and healthy pregnancy groups, respectively. The median gravida values of the groups were 3 (1–6), 2 (1–10), and 3 (1–5), respectively. The median parity values of the groups were 1 (0–3), 1 (0–5), and 1.5 (0–4); median abortion values were 0 (0–3), 0 (0–7), and 0 (0–2); the median living values were 1 (0–3), 1 (0–5), and 1.5 (0–4), respectively. None of the patients had alcohol use, and smoking was present in 7 (23.3%), 3 (10.0%), and 5 (16.7%) patients in the missed abortion, abortus imminens, and healthy pregnancy groups, respectively. There was no statistically significant difference among the groups for parameters of age, gravida number, parity number, abortion number, BMI, and systolic and diastolic blood pressures. The median gestational week and mean CRL values of the study population were 9 (6–13) weeks and 22.01 ± 14.25 mm respectively. There was no statistically significant difference among groups for parameters of gestational week and CRL measurement.

The whole blood count, thyroid function, and liver function tests, INR, and MFG-E8 levels of the study population are presented in Table 2. No statistically significant difference was found among the groups for parameters of WBC count, lymphocyte count, hemoglobin level, hematocrit percentage, platelet count, PDW, MPV, TSH, AST, and ALT values. Despite being between normal ranges, the mean neutrophil count and INR values of the MA, TA, and HP groups showed statistically significant difference (p < 0.05). The pairwise comparison of groups is presented in Table 3. It was found that the mean neutrophil count in the MA group was statistically significantly lower than the HP group (p = 0.013). It was observed that the highest mean value of the INR was in the TA group and it was statistically significantly higher than that of the HP group (p = 0.008).

The mean MFG-E8 values of MA, TA, and HP groups were 270 ± 152.3, 414.7 ± 236.7, and 474 ± 222.5 ng/mL, respectively (p = 0.001) (Table 2). It was found that the mean MFG-E8 level of the MA group was statistically significantly lower than those of the other two groups (p = 0.016 and p = 0.000). There was no statistically significant difference between TA and HP groups (p = 0.217) (Table 3).

When all of the patients included in the study were evaluated, a negative correlation was found between AST (r = −0.398, p < 0.001), ALT (–0.243, p < 0.05) and lymphocyte (r = −0.225, p < 0.05) and MFGE8. A negative correlation was found between ALT (r = −0.390, p < 0.05), CRL (r = −0.472, p < 0.001), CRL week (r = −0.575, p < 0.05) and MFGE8 in the missed abortion group.

A negative correlation was found only between AST (r = −0.382, p < 0.05) and MFGE8 in the abortion imminens group. In the healthy pregnancy group, a negative correlation was found between age (r = −0.408, p < 0.05) and AST (r = −0.367, p < 0.05) and MFGE8.

The mean PLR was found to be 133.24 ± 46.34 in all patients. The mean PLR was found to be 127.10 ± 44.68, 141.88 ± 53.55, and 130.75 ± 40.05 in missed abortion, abortus imminens, and healthy pregnant groups, respectively. The mean NLR was found to be 3.54 ± 2.00 in all patients. The mean NLR was found to be 2.98 ± 2.08, 3.37 ± 1.36, and 4.28 ± 2.28 in missed abortion, abortus imminens, and healthy pregnant groups, respectively. A positive correlation (r = 0.416, p < 0.001) was found between NLR and PLR in all patients, but no correlation was found between these values and MFGE8 (p > 0.05). When the missed abortion, abortion imminens, and healthy pregnant groups were evaluated separately, there was also a positive correlation between NLR and PLR (r = 0.602, p < 0.001, r = 0.565, p < 0.05 and r = 0.565, p < 0.05, respectively). However, no correlation was found between MFGE8 and PLR or NLR values in all patients or in any of the groups when evaluated separately (p > 0.05). SII median (minimum-maximum) values of all patients, missed abortion, abortus imminens, and healthy pregnant groups were 748.3 (215.4–4835.3), 651.6 (264.1–4835.3), 811.1, (376.5–2126.9), and 848.9 (215.4–4713.8), respectively. For missed abortion group (r = −0.340, p > 0.05), abortion imminens group (r = 0.027, p > 0.05), or healthy pregnant group (r = −0.09, p > 0, 05), no statistically significant correlation was found between SII and MFGE8.

In the ROC analysis, MFG-E8 values below 301.3 ng/mL have a predictive value (AUC = 0.736, p < 0.001) with 70.0% sensitivity and 65.5% specificity to distinguish missed abortion cases from live intrauterine pregnancies (threatened abortion and healthy pregnancy).

The pregnancy outcomes of TA and HP groups are presented in Table 4. There was no significant difference between TA and HP groups for parameters of neonatal birth weight, delivery mode, apgar 1 and 5 scores, and pregnancy outcome. There was no correlation between maternal serum MFG-E8 level and neonatal birth weight and Apgar scores.

## 4. Discussion

According to our knowledge, this the first study investigating maternal serum MFG-E8 levels of first-trimester pregnant women suffering from missed abortion and threatened abortion. We observed significantly decreased maternal serum MFG-E8 levels in missed abortion cases compared to the gestational week-, age-, and BMI-matched pregnant women carrying live fetus. MFG-E8 expression in the female reproductive system was first described in 2003 by Rehman et al. In the study of Rehman et al., microarray analysis showed that MFG-E8 expression was 3.9 times higher in the myometrium of pregnant women compared to no-pregnant women, and it was suggested that MFG-E8 has a pregnancy-related role [21]. MFG-E8 has also recently been associated with the implantation process. Researchers compared the gene expression profile of MFG-E8 during the early and middle luteal phases, and it was found that MFG-E8 expression increased in the human endometrium during the implantation process [14,15].

The endometrium undergoes cyclical changes in preparation for embryo implantation with expression of multiple proteins. The endometrium is maximally suitable for attachment during the implantation process; which includes embryo apposition, adhesion and invasion [22]. One of these proteins is αvβ3 integrin which is thought to be associated with embryo attachment [23]. Integrin αvβ3 is the receptor of MFG-E8, a vitronectin receptor, and it is a type of integrin that interacts with various ligands such as vitronectin, fibronectin, osteopontin, metalloproteinase-2 [24]. It is involved in cell migration, tumor invasion, bone resorption, angiogenesis, and immune response [25]. This adhesion molecule is expressed by endometrial epithelial cells after day 6 of the luteal phase and is thought to be impaired in women with implantation failure [26, 27]. In the study of Elnaggar et al., αvβ3 integrin levels were found to be statistically significantly lower in the endometrium of infertile women compared to fertile ones [28].

In the study of Franchi et al., it was shown that MFG-E8 protein is expressed during the implantation process and mostly in endometrial epithelial cells of ovulatory women; and αvβ3 integrin, which is the MFG-E8 receptor, is present in epithelial and stromal compartments of endometrium. In the study, it was stated that MFG-E8 is a glycoprotein with a key role in the regulation of endometrial functions with increased expression towards the end of the implantation window [29]. αvβ3 integrin is expressed not only in the endometrium but also in the embryo [30]. In the study of Sutherland et al., integrin αvβ3 was found to be expressed in mouse embryos and in the study of Campbell et al., it was found to be expressed in human embryos [31, 32]. The researchers have shown that MFG-E8 protein is highly expressed in human chorionic villi (both cytotrophoblasts and syncytiotrophoblasts) and mouse implantation sites during all trimesters of pregnancy [15, 33]. In another study, recombinant MFG-E8 was found to modulate endometrial endothelial cell proliferation and adhesion in vitro [34].

In the study by Schmitz et al., an in vitro assay mimicking human implantation was created using a well-differentiated endometrial adenocarcinoma cell line and choriocarcinoma human trophoblast cells. To see the results of blocking the action of MFG-E8 and its receptor αvβ3, cell lines were pretreated with different concentrations of antibodies against these proteins prior to the attachment assay. Pretreatment of cells with anti-MFG-E8 antibody and antiintegrin αvβ3 appeared to cause significant dose-dependent inhibition of adhesion [35].

In the in vitro study by Yu et al. using human endometrial epithelial cells and mouse blastocysts, it was found that endometrial MFG-E8 modulates transforming growth factor-beta (TGF-β)-induced epithelial mesenchymal transition (EMT) in human endometrial cells and promotes embryo attachment and early invasion. EMT plays an important role in embryo development and tissue repair. TGF-β causes EMT junction changes and promotes embryo attachment. According to the study, when MFG-E8 is deactivated in endometrial epithelial cells, the EMT process and TGF-β-triggered interaction between embryo and human endometrial epithelial cells are inhibited. In the light of these findings, it was stated that MFG-E8 has an important role in trophoblast attachment, early invasion, and endometrial remodeling [36]. All these studies strongly suggest that endometrial MFG-E8 plays an important role in physiological conditions during the menstrual endometrial remodeling and implantation process and dysfunctions in its expression may be associated with pathological conditions in early pregnancy.

In our study, the mean values of maternal serum MFG-E8 were 270.03 ± 152.28 ng/mL in the MA group, 414.73 ± 236.73 ng/mL in the TA group, and 474.03 ± 222.53 ng/mL in the HP group. It was observed that MFG-E8 values were lower in the MA and TA groups compared to that of the HP group. It was determined that the mean MFG-E8 value was at the lowest level in the MA group and it was statistically significantly lower than those of both the TA group and the HP group (p < 0.05). There was no statistically significant difference in the TA group compared to HP group (p > 0.05). In addition, in the ROC analysis, it was found that MFG-E8 values below 301.3 ng/mL could distinguish missed abortion cases from pregnant women with live intrauterine pregnancy with 70% sensitivity and 65.5% specificity.

There are limited studies in the literature about MFG-E8 levels and complicated pregnancies. Li et al. observed increased plasma MFG-E8 levels in pregnant women suffering from gestational diabetes mellitus compared to healthy pregnant women [37]. Aydin et al. reported significantly increased plasma MFG-E8 levels in the preeclamptic pregnant women compared to the healthy pregnant women [38].

There are some limitations in our study. The first of these is the limited study population number. Another limitation is that the placental level and expression of MFG-E8 could not be studied. The third limitation is that we could not investigate the umbilical cord MFG-E8 levels and alterations in maternal serum levels among trimesters.

In conclusion, first-trimester maternal serum MFG-E8 level can distinguish missed abortion cases from pregnant women with healthy intrauterine pregnancy. New studies with larger patient groups to support the findings of our study will be very valuable in terms of using MFG-E8 in estimating early pregnancy prognosis and predicting and diagnosing missed abortion development.

## Figures and Tables

**Table 1:** Demographic and clinical characteristics of the whole study population.

	Missed abortion (n = 30)	Threatened abortion (n = 30)	Healthy pregnancy (n = 30)	p-value
**Age (years)**	29.83 ± 6.21	29.00 ± 4.48	29.23 ± 5.24	0.888
**Gravida**	3 (1–6)	2 (1–10)	3 (1–5)	0.588
**Parity**	1 (0–3)	1 (0–5)	1.5 (0–4)	0.538
**Abortion**	0 (0–3)	0 (0–7)	0 (0–2)	0.084
**Gestational week**	9 (7–13)	9(8–13)	9 (6–13)	0.070
**CRL (mm)**	20.66 ± 16.10	21.87 ± 12.76	24.42 ± 11.80	0.080
**BMI (kg/m** **^2^** **)**	26.70 ± 4.84	25.78 ± 3.25	25.53 ± 5.39	0.758
**BP-systolic (mmHg)**	106.50 ± .62	109.00 ± 10.61	105.00 ± 9.94	0.213
**BP-diastolic (mmHg)**	69.33 ± 9.89	67.66 ± 8.58	66.10 ± 6.63	0.382

Note: Data are presented as mean ± standard deviation and median (minimum-maximum).

BMI: Body mass index, BP: Blood pressure, CRL: Crown-rump length, mm: millimeter.

**Table 2:** Comparison of the blood parameters and MFG-E8 of the study population.

	Missed abortion (n = 30)	Threatened abortion (n = 30)	Healthy pregnancy (n = 30)	p-value
**WBC (10^3^/**μL**)**	9.03 ± 4.73	9.08 ± 2.66	9.70 ± 3.74	0.352
**Neutrophil count (10^3^/**μL**)**	6.49 ± 2.25	6.22 ± 2.14	7.42 ± 3.59	**0.044**
**Lymphocyte count (10^3^/**μL**)**	2.26 ± 0.79	1.95 ± 0.57	1.80 ± 0.41	0.540
**Hemoglobin (g/dL)**	12.62 ± 1.23	12.25 ± 1.65	12.16 ± 1.12	0.388
**Hematocrit (%)**	37.93 ± 3.42	36.75 ± 3.92	36.35 ± 2.97	0.190
**Platelet (10^3^/**μL**)**	260.56 ± 50.97	258.43 ± 63.99	229.43 ± 67.54	0.095
**PDW (fL)**	16.11 ± 0.53	16.13 ± 0.55	16.00 ± 1.23	0.246
**MPV (fL**)	10.37 ± 0.87	10.92 ± 1.75	10.13 ± 0.99	0.202
**TSH (uIU /mL)**	1.86 ± 1.23	1.90 ± 1.04	1.54 ± 0.79	0.434
**AST (IU/L)**	18.90 ± 5.35	20.26 ± 8.21	17.77 ± 6.82	0.140
**ALT (IU/L)**	16.80 ± 8.56	14.83 ± 10.80	13.88 ± 8.03	0.113
**INR**	0.92 ± 0.07	0.95 ± 0.11	0.87 ± 0.10	**0.018**
**MFG-E8 (ng/mL)**	270.03 ± 152.28	414.73 ± 236.73	474.03 ± 222.53	**0.001**

Note: Data are presented as mean ± standard deviation and median (minimum-maximum).

WBC: White blood cell. PDW: Platelet distribution width. MPV: Mean platelet volume. TSH: Thyroid stimulating hormone. AST: Aspartate aminotransferase. ALT: Alanine aminotransferase. INR: International normalized ratio. MFG-E8: Milk fat globule-epidermal growth factor 8.

**Table 3:** Pairwise group comparisons of neutrophil count, INR, and MFG-E8.

	MA vs TA p-value	TA vs HP p-value	MA vs HP p-value
**Neutrophil count (10^3^/**μL**)**	0.139	0.311	**0.013**
**INR**	0.239	**0.008**	0.055
**MFG-E8 (ng/mL)**	**0.016**	0.217	**0.000**

Note: MA= Missed abortion, TA= Threatened abortion, HP = Healthy pregnancy, INR: International normalized ratio.

**Table 4:** Pregnancy outcomes of the TA and HP groups.

	TA group (n = 30)	HP group (n = 30)	p-value
**Delivery mode (%)**			
Vaginal	20	23.3	0.52
Cesarean sectio	50	70	
Abortion	30	6.7	
**Neonatal birth weight (gr)**	3144 ± 649	3164 ± 501	0.49
**Delivery week**	38(31–40)	38 (34–41)	0.23
**APGAR 1 score**	8 (0–10)	9 (7–9)	0.58
**APGAR 5 score**	9 (0–10)	10 (7–10)	0.24
**Pregnancy related complications (%)**			
Ablatio placenta	3.3	-	0.45
Preeclampsia	3.3	-	
İntrauterine exitus	3.3	-	
GDM	-	6.7	
Oligohydramnios	-	3.3	
PPROM	-	3.3	

Note: Values are presented as mean ± SD or median (minimum-maximum) and percentage.TA: Threatened abortion, HP: HEALTHY pregnant, GDM: Gestational diabetes mellitus,

PPROM: Premature preterm rupture of membranes.
